# Modified Brief Cognitive Screening Battery - Indonesian Version: cross-cultural adaptation and normative data based on demographic factors in North Sumatra, Indonesia

**DOI:** 10.3389/fneur.2023.1306356

**Published:** 2024-01-15

**Authors:** Fasihah Irfani Fitri, Lorina Naci, Yuda Turana, Aldy Safruddin Rambe, Dina Nazriani, Ricardo Nitrini, Paulo Caramelli

**Affiliations:** ^1^Global Brain Health Institute, Trinity College Dublin, Dublin, Ireland; ^2^Department of Neurology, Faculty of Medicine, Universitas Sumatera Utara, Medan, Indonesia; ^3^School of Psychology, Trinity College Dublin, Dublin, Ireland; ^4^School of Medicine and Health Sciences, Atma Jaya Catholic University of Indonesia, Jakarta, Indonesia; ^5^Faculty of Psychology, Universitas Sumatera Utara, Medan, Indonesia; ^6^Department of Neurology, University of São Paulo Medical School, São Paulo, Brazil; ^7^Behavioral and Cognitive Research Group, Faculdade de Medicina, Universidade Federal de Minas Gerais, Belo Horizonte, Brazil

**Keywords:** cognitive, dementia, screening, Indonesia, older adult

## Abstract

**Introduction:**

Key component of early detection of dementia is a brief and culturally appropriate cognitive screening tool. This study aimed to perform a cultural adaptation of the Brief Cognitive Screening Battery (BCSB) and to obtain normative data from the older adult population.

**Methods:**

Cross-cultural adaptation process to develop BCSB-INA was performed. This was followed by a feasibility study from community dwelling older adults from several urban and rural areas in North Sumatra, Indonesia.

**Results:**

The BCSB-INA was generally well understood and showed not much discrepancy in translation from the original version. There were differences in semantic and phonemic fluency and CDT based on years of education, but no difference was found on other domain, including the delayed recall of the FMT. The battery was more influenced by age than education.

**Discussion:**

The BCSB-INA is culturally appropriate and feasible to be used in population with heterogenous educational background in Indonesia.

## 1 Introduction

The rise in life expectancy increases the likelihood of developing dementia, given that age stands as the most recognized risk factor for this condition. The expansion of this aging population is most pronounced and rapid in low- and middle-income countries (LMICs), including Indonesia. Early diagnosis is important for identifying potentially reversible causes, improving symptom management and quality of life, and facilitating future planning. However, it is estimated that around 90% of people with dementia (PWD) are not diagnosed in LMICs, even though timely diagnosis can lead to better management and improve the quality of life of PWD and their families (1, 2). In the absence of a cure for most causes of dementia, reducing the future costs of dementia care may be best attained by timely diagnosis followed by earlier intervention, to maintain functional independence (3). Crucial to early detection is the cognitive screening in dementia high-risk groups, by using brief yet comprehensive cognitive assessment instruments (4). One of the key barriers to assessment and diagnosis of dementia is the inadequate linguistic and cultural validation of cognitive assessment tools, along with other issues including negative perceptions surrounding dementia, limited patient involvement in and availability of healthcare services, limited dementia-capable workforce, conflicting priorities within the healthcare system and inadequate financial support (5, 6).

The most widely used tools for screening of cognitive impairment are the Mini Mental State Examination (MMSE) and the Montreal Cognitive Assessment (MoCA) (7–10) which are primarily verbal-based, and derived from the translation of English, which present challenges when applied in the multilingual, and educationally diverse Indonesian population. One systematic review of cognitive assessment tools in Asia showed that the performance of the MMSE has been widely reported to be influenced by age and education as well as language, ethnicity, and cultural differences (10) and showed low sensitivity for detecting mild cognitive impairment (MCI) (11). Indonesians with a higher level of education were more appropriate for using the MMSE than those with lower education (12). The MoCA is also strongly influenced by education (11) and the items require adaptation before they are applicable to other cultures, such as picture naming and word lists. Moreover, the low literacy rates in Asian countries, including Indonesia, might lead to an educational bias that may result in an overestimation of the prevalence of dementia and lead to inappropriate referral or diagnosis (10).

Although the translation of these cognitive instruments is clearly needed and helpful in their administration to patients, it engenders some gaps and meaning or content, which is a big challenge for accurate assessment of the Indonesian population. Therefore, there is an immediate need for a simple, language-neutral, visual-based test, that is also culturally appropriate and can be used in daily clinical practice. A brief cognitive screening tool that is free from translation bias and can be used by general practitioners will facilitate cognitive screening and lead to better referrals, and subsequently better diagnosis and care. This study aimed to develop a simple and culturally validated tool by doing a cultural adaptation of the Brief Cognitive Screening Battery Test (BCSB)—a test shown to be suitable in rural and urban populations with low educational levels. The original BCSB included animal verbal fluency, the clock drawing test (CDT), and the figure memory test (FMT).

The FMT includes assessments for naming, incidental, immediate memory, learning, delayed recall, and recognition. The pictures consisted of 10-line drawings (shoe, house, comb, key, airplane, turtle, book, spoon, tree, and bucket) (13, 14). The second aim was to assess its feasibility in community-dwelling older adults in several rural and urban areas in North Sumatra, Indonesia, and to obtain normative data from the population.

## 2 Methods

This was a cross-sectional study that consisted of two major steps. The first one was to construct the cognitive tool and the second was to assess its feasibility in community-dwelling older adults.

### 2.1 The Brief Cognitive Screening Battery

The BCSB consists of presenting a sheet of paper with 10 simple black and white drawings. The figures are named by the subjects (Naming score), who are then asked to recall each drawing immediately, by listing them verbally, without having been told that the figures should be memorized (Incidental Memory score). Subsequently, the figures are displayed again and the subjects are asked to memorize them for 30 s, after which they should be recalled (Immediate Memory score). The same procedure is then repeated to obtain the Learning score (or encoding). Then, after the Verbal Fluency test (animals in 1 min) and the clock drawing test (CDT), which are interference tests, subjects are asked to recall the figures shown previously (Delayed Recall score). Finally, a sheet of paper with the 10 target figures, mixed with 10 new distracting figures, is presented and the subjects are asked to recognize those figures displayed originally (Recognition score). Scoring on these subtests ranges from 0 to 10 points (except for the Verbal Fluency test) (14).

### 2.2 Development and cultural adaptation of the cognitive tool

The BCSB was culturally validated using ISPOR Principles of Good Practice for the cross-cultural adaptation process for the patient-reported outcomes measures (15). This included:

1. Preparation: including contacting and requesting permission from the author of the original BCSB.
2. Forward translation: the original instruction of BCSB was first translated into Bahasa Indonesia by the Language Center in Universitas Sumatera Utara and from an official English sworn translator independently.
3. Reconciliation: comparing and merging the two forward translations into a single forward translation.
4. Back translation: translation of the new English version back into Bahasa Indonesia. This was done by another independent translator.
5. Back translation review: compare the back-translated versions of the instrument with the original to investigate discrepancies.
6. Harmonization: comparison of the back-translated version with the original instrument to ensure conceptual equivalence between the English and the Bahasa Indonesia versions.
7. Cognitive debriefing: testing the instrument on a small group of people in order to test alternative wording and to check the understandability, interpretation, and cultural relevance of the translation. We interviewed 20 people for this cognitive pre-testing.
8. Review of cognitive debriefing results and finalization: comparison of the older adults' interpretation of the translation with the original version to highlight and amend discrepancies.
9. Proofreading: final review of the translation to highlight and correct any typographic, grammatical, or other errors.
10. Final report: report written at the end of the process documenting the development of each translation.

Translation was applied to instruction and interpretation/scoring only, because this is a visual-based test. The Indonesian version of BCSB (BCSB-INA) was then called The *Instrumen Skrining Kognitif Ringkas di Indonesia (INA-SKRin)*, which is the literal translation of BCSB, to make it easier to comprehend when administered to the subjects. The BCSB-INA also included the phonemic fluency test. For the phonemic fluency test the letter “S” was used as it has been widely used, for example in MoCA-INA. The subjects will be instructed to name as many words as possible (excluding name, city, and place) that begin with this letter in 1 min (9).

### 2.3 Feasibility/pilot study

This stage included administering the tool to community-dwelling older adults in urban and rural areas to assess its cultural appropriateness and time of completion in several areas in North Sumatra, Indonesia. North Sumatra is a multi-ethnic province with at least 11 ethnic groups, currently divided into 25 regencies and eight autonomous cities. The official language is Bahasa Indonesia, but there are at least five local languages that are also commonly used, especially in rural areas (16). The population represented urban and rural groups, various levels of education and literacy backgrounds, and different ethnicities. This stage included:

1. Recruitment and training of the interviewers

Interviewers were recruited with the following criteria: a minimum of 12 years of education, had health background of education, good health condition, good verbal communication skills, and the ability to work in a team. Formal training of the interviewers included the following topics: general standard operating procedure, the tool and how to administer it, selecting participants, informed consent, and data management.

2. Participants recruitment

Participants were recruited in three primary healthcare facilities in each area: Kota Matsum, Selayang, and Helvetia in Medan, the capital city of North Sumatra, representing the urban area, and Perbaungan, Desa Pon, and Tanjung Beringin in Serdang Bedagai representing the rural area. Simple random sampling was used to select the districts in Medan and Serdang Bedagai. We included older adults aged 60 years or older at the date of consent, with stable medical conditions, who speak Bahasa Indonesia and who lived within the defined sampling areas who attended the primary healthcare facilities. Participants were asked to provide an informant to confirm the demographic data and existing medical conditions. We excluded participants with significant vision and hearing impairment that could interfere with the cognitive assessment.

### 2.4 Procedures

Written informed consent was obtained from all participants. Each participant was assessed by a trained interviewer at the primary healthcare facility. The battery was administered as a paper and pencil test. Another interviewer interviewed the Ascertain Dementia, 8-item informant questionnaire (AD8) Indonesian version (17) to the family or partner to confirm that the participants had no known history of dementia or change in cognitive function and were in a stable medical condition.

### 2.5 Statistical analysis

The data were analyzed statistically using Windows Statistical Product and Science Service (SPSS) version 22. Descriptive analysis was used to present demographic data and scores of cognitive tests. Non-parametric tests were used due to the non-normal distribution of the data. Comparative analysis using Mann-Whitney or Kruskal-Wallis was used to analyze the difference in cognitive scores between sex, age, education, and area of living. The level of significance was set at 0.05.

### 2.6 Ethical consideration

All procedures contributing to this work comply with the ethical standards of the relevant national and institutional committees on human experimentation and with the Helsinki Declaration. Ethical approval was obtained from the Universitas Sumatera Utara (approval number 498/KEP/FK USU/2022). All subjects or their legally authorized representatives provided written informed consent.

## 3 Results

### 3.1 Development and cultural adaptation of the cognitive tool

To develop the Indonesian version of the BCSB we followed the ISPOR Principles of Good Practice and the World Health Organization recommendations for the cross-cultural adaptation of patient report outcome. During the process, expert meetings were continuously held at different stages (reconciliation and harmonization). The experts consisted of two senior consultant neurologists with extensive experience working with dementia patients in North Sumatra, one behavioral neurologist with experience in clinical and research in dementia and cognitive assessment from the Indonesian Neurology Association and one consultant of language and culture. We also obtained inputs from the original authors of BCSB.

Since it is primarily a visual-based tool, the results of the translation process did not pose any issues. The cognitive interview process showed that the translated instruction was generally well understood by the participants. The length of assessment was < 10 min. We kept the original pictures as they are universal and were generally recognized by the participants. There were several items with more than one name (e.g., bucket was formally translated as *ember* in Bahasa Indonesia, but it is also known as *timba* as local term in North Sumatera). For this case, both names were considered correct. The BCSB-INA was easy to administer and generally well understood.

### 3.2 Feasibility/pilot study

We included 211 participants, consisting of 168 (79.6%) females and 43 (20.4%) males. The mean age was 67.8 ± 6.1 years, ranged from 60 to 92 years (median 67 years). The mean years of education was 8.1 ± 4.1 years, ranged from 0 to 18 years (median 8 years). The subject characteristics is shown in Table 1. The scores of BCSB-INA (INA-SKRin) are shown in Table 2. The normative data based on demographic characteristics including sex, age, education and area of living are shown in Tables 3–6. Based on sex, female subjects performed better on incidental memory while male subjects performed better on semantic fluency test (Table 3). Subjects living in urban area (Medan) had significantly better performances on learning, semantic and phonemic fluency tests and CDT compared to those living in rural area (Serdang Bedagai), as shown in Table 4. Comparison between age groups showed that older adults aged 60–74 years performed better on the following domains: immediate memory, learning, recall, semantic and phonemic fluency tests compared to those aged ≥75 (Table 5). Comparison among levels of education showed that older adults with higher education had better performance only on semantic and phonemic fluency tests and CDT. Spearman correlation tests showed that the subtests of the battery were correlated more with age and less with education, as shown in Table 7.

**Table 1:** Basic demographic characteristics among 211 older adults.

**Variable**	***n* = 211**
**Sex**, ***n*** **(%)**	
Female	168 (79.6)
Male	43 (20.4)
**Age, years**	
Mean (SD)	67.8 (6.1)
Median (min–max)	67 (60–92)
**Age group**, ***n*** **(%)**	
60–74 years	181 (85.8)
≥75 years	30 (14.2)
**Education**, ***n*** **(%)**	
None	1 (0.5)
Elementary school	99 (46.9)
Junior high school	44 (20.9)
Senior high school	49 (23.2)
College/university	18 (8.6)
**Education, years**	
Mean (SD)	8.1 (4.1)
Median (min–max)	8 (0–18)
**Education**, ***n*** **(%)**	
0 year	2 (0.95)
1–4 years	39 (18.48)
5–8 years	65 (30.81)
9–11 years	40 (18.95)
≥12 years	65 (30.81)

**Table 2:** Cognitive function characteristics of BCSB-INA (INA-SKRin) among 211 older adults.

**BCSB-INA subtests**	**Mean**	**SD**	**Median**	**Minimum**	**Maximum**
Naming	9.9	0.7	10	3	10
Incidental memory	6.8	1.9	7	1	10
Immediate memory	7.8	1.8	8	0	10
Learning	8.3	1.7	9	2	10
Recall	7.7	2	8	1	10
Recognition	9.6	1.1	10	3	10
Clock drawing test	4.2	2.3	4	1	9
Semantic fluency	12.5	4	12	3	23
Phonemic fluency	6	4	5	0	20

BCSB, Brief Cognitive Screening Battery - Indonesian Version; INA SKRin, Instrumen Skrining Kognitif Ringkas-Indonesia; SD, standard deviation.

**Table 3:** Scores of the BCSB-INA (INA-SKRin) according to sex.

	**Male (43)**	**Female (168)**	***p-*value**		**Male (43)**	**Female (168)**	***p-*value**		**Male (43)**	**Female (168)**	***p-*value**
**Naming**			0.712	**Learning**			0.318	**CDT**			0.943
Mean	9.91	9.85		Mean	7.93	8.33		Mean	4.23	4.24	
SD	0.366	0.804		SD	2.02	1.59		SD	2.43	2.28	
Median	10	10		Median	8	9		Median	4	4	
Minimum	8	3		Minimum	2	3		Minimum	1	1	
Maximum	10	10		Maximum	10	10		Maximum	9	9	
**Incidental memory**			0.038	**Semantic fluency**			0.043	**Delayed recall**			0.271
Mean	6.26	6.92		Mean	13.5	12.2		Mean	7.44	7.76	
SD	1.99	1.85		SD	4.38	3.85		SD	1.93	1.96	
Median	6	7		Median	14	12		Median	8	8	
Minimum	1	1		Minimum	3	4		Minimum	1	1	
Maximum	10	10		Maximum	22	23		Maximum	10	10	
**Immediate memory**			0.142	**Phonemic fluency**			0.639	**Recognition**			0.745
Mean	7.42	7.95		Mean	6.65	5.92		Mean	8.86	8.98	
SD	2.11	1.7		SD	4.87	3.62		SD	2.37	2.05	
Median	8	8		Median	6	5		Median	10	10	
Minimum	0	2		Minimum	1	1		Minimum	0	1	
Maximum	10	10		Maximum	20	18		Maximum	10	10	

BCSB, Brief Cognitive Screening Battery - Indonesian Version; INA SKRin, Instrumen Skrining Kognitif Ringkas-Indonesia; SD, standard deviation.

**Table 4:** Scores of the BCSB-INA (INA-SKRin) according to location (urban vs. rural).

	**Medan 107**	**Sergei 104**	***p-*value**		**Medan**	**Sergei**	***p-*value**		**Medan**	**Sergai**	***p-*value**
**Naming**			0.248	**Learning**			0.014	**CDT**			< 0.001
Mean	9.79	9.93		Mean	8.5	7.99		Mean	4.89	3.56	
SD	0.962	0.376		SD	1.62	1.72		SD	2.19	2.22	
Median	10	10		Median	9	8		Median	5	3	
Minimum	3	7		Minimum	2	2		Minimum	1	1	
Maximum	10	10		Maximum	10	10		Maximum	9	9	
**Incidental memory**			0.468	**Semantic fluency**			0.007	**Delayed recall**			0.244
Mean	6.73	6.84		Mean	13.3	11.7		Mean	7.83	7.56	
SD	1.74	2.04		SD	4.24	3.54		SD	1.95	1.96	
Median	7	7		Median	13	12		Median	8	8	
Minimum	1	1		Minimum	4	3		Minimum	1	1	
Maximum	10	10		Maximum	23	22		Maximum	10	10	
**Immediate memory**			0.335	**Phonemic fluency**			0.007	**Recognition**			0.110
Mean	7.95	7.73		Mean	7.22	4.88		Mean	9.16	8.74	
SD	1.77	1.82		SD	4.24	3.54		SD	1.86	2.33	
Median	8	8		Median	13	12		Median	10	10	
Minimum	0	3		Minimum	4	3		Minimum	1	0	
Maximum	10	10		Maximum	23	22		Maximum	10	10	

BCSB, Brief Cognitive Screening Battery - Indonesian Version; INA SKRin, Instrumen Skrining Kognitif Ringkas-Indonesia; SD, standard deviation.

**Table 5:** Scores of the BCSB-INA (INA-SKRin) stratified by age.

	**60–74 years**	**≥75 years**	***p-*value**		**60–74 years**	**≥75 years**	***p-*value**		**60–74 years**	**≥75 years**	***p-*value**
**Naming**			0.277	**Learning**			0.001	**CDT**			0.781
Mean	9.9	9.8		Mean	8.4	7.3		Mean	4.3	4.1	
SD	0.7	0.9		SD	1.6	1.7		SD	2.4	2.1	
Median	10	10		Median	9	7		Median	4	4	
Minimum	3	5		Minimum	2	4		Minimum	1	1	
Maximum	10	10		Maximum	10	10		Maximum	9	7	
**Incidental memory**			0.092	**Semantic fluency**			0.021	**Delayed recall**			0.021
Mean	6.9	6.3		Mean	12.8	11		Mean	7.8	6.9	
SD	1.9	1.9		SD	4.1	2.6		SD	1.9	2.2	
Median	7	6		Median	12	11		Median	8	7	
Minimum	1	2		Minimum	3	7		Minimum	1	1	
Maximum	10	9		Maximum	23	16		Maximum	10	10	
**Immediate memory**			0.015	**Phonemic fluency**			0.031	**Recognition**			0.080
Mean	7.9	7.2		Mean	6.2	4.6		Mean	9.6	9.2	
SD	1.8	1.6		SD	4	3.3		SD	0.9	1.5	
Median	8	7		Median	6	5		Median	10	10	
Minimum	0	3		Minimum	0	0		Minimum	4	3	
Maximum	10	10		Maximum	20	15		Maximum	10	10	

BCSB, Brief Cognitive Screening Battery - Indonesian Version; INA SKRin, Instrumen Skrining Kognitif Ringkas-Indonesia; CDT, clock drawing test; SD, standard deviation.

**Table 6:** Scores of the BCSB-INA (INA-SKRin) stratified by education.

	**0**	**1–4**	**5–8**	**9–11**	**≥12 years**	***p-*value**		**0**	**1–4**	**5–8**	**9–11**	**≥12 years**	***p-*value**		**0**	**1–4**	**5–8**	**9–11**	**≥12 years**	***p-*value**
	***N*** = **2**	***N*** = **39**	***N*** = **65**	***N*** = **40**	***N*** = **65**			***N*** = **2**	***N*** = **39**	***N*** = **65**	***N*** = **40**	***N*** = **65**			***N*** = **2**	***N*** = **39**	***N*** = **65**	***N*** = **40**	***N*** = **65**	
**Naming**						0.656	**Learning**						0.147	**CDT**						< 0.001
Mean	10	9.79	9.92	9.9	9.8		Mean	9	7.82	7.95	8.5	8.63		Mean	4.5	2.95	4	4.5	5.06	
SD	0	0.923	0.407	0.632	0.922		SD	0	2.08	1.79	1.41	1.4		SD	2.12	1.89	2.38	1.97	2.3	
Median	10	10	10	10	10		Median	9	8	8	9	9		Median	4.5	3	3	4	5	
Minimum	10	5	7	6	3		Minimum	9	2	2	5	4		Minimum	3	1	1	1	1	
Maximum	10	10	10	10	10		Maximum	9	10	10	10	10		Maximum	6	7	9	8	9	
**Incidental memory**						0.764	**Semantic fluency**						0.002	**Delayed recall**						0.397
Mean	7	6.87	6.89	6.72	6.65		Mean	13.5	10.6	12.2	12.8	13.8		Mean	8.5	7.21	7.57	8.05	7.88	
SD	1.41	2.12	1.94	1.77	1.83		SD	0.707	3.23	3.77	3.68	4.39		SD	2.12	2.3	2.11	1.88	1.57	
Median	7	7	7	7	7		Median	13.5	10	12	12	14		Median	8.5	7	8	8	8	
Minimum	6	1	1	3	2		Minimum	13	5	5	7	3		Minimum	7	1	1	2	4	
Maximum	8	10	10	10	10		Maximum	14	18	22	23	23		Maximum	10	10	10	10	10	
**Immediate memory**						0.915	**Phonemic fluency**						< 0.001	**Recognition**						0.415
Mean	7.5	7.64	7.91	8	7.82		Mean	2.5	3.69	4.78	5.53	9.23		Mean	9	8.28	8.91	9.13	9.29	
SD	0.707	2.11	1.67	1.77	1.79		SD	0.707	2.69	2.44	2.79	4.39		SD	1.41	2.93	2.06	1.98	1.58	
Median	7.5	8	8	8.5	8		Median	2.5	3	5	5	9		Median	9	10	10	10	10	
Minimum	7	0	3	3	2		Minimum	2	1	1	1	1		Minimum	8	0	1	1	2	
Maximum	8	10	10	10	10		Maximum	3	10	11	13	20		Maximum	10	10	10	10	10	

BCSB, Brief Cognitive Screening Battery - Indonesian Version; INA SKRin, Instrumen Skrining Kognitif Ringkas-Indonesia; CDT, clock drawing test; SD, standard deviation.

**Table 7:** Correlations of INA Skrin with age and education.

	**Naming**	**Incidental memory**	**Immediate memory**	**Learning**	**Recall**	**Recognition**	**CDT**	**Semantic**	**Phonemic**
Age	0.013^*^	−0.107^*^	−0.101^*^	−0.146^**^	−0.109^*^	−0.118^*^	−0.068^*^	−0.105^*^	−0.159^**^
Education	−0.063	−0.081	0.039^*^	0.181	0.100	0.076^*^	0.332	0.286	0.539

Spearmen bi-variate correlations ^*^p < 0.05 ^**^p < 0.01.

INA Skrin, Instrumen Skrining Kognitif Ringkas-Indonesia; CDT, clock drawing test.

## 4 Discussion

The current study was conducted to translate and culturally adapt the BCSB to develop and Indonesian version of the battery and to obtain normative data from the older adult population in North Sumatra.

### 4.1 Development and cultural adaptation of the cognitive tool

Cultural adaptation of a cognitive tool refers to modifying a cognitive tool to better suit the cultural context and needs of a specific group or community. This process involves considering cultural norms, values, language, and practices to ensure the tool's effectiveness and relevance within that particular cultural setting. It may involve incorporating local examples, and idioms, or addressing potential biases that could impact the use in the target culture. The goal is to enhance the tool's usability and acceptance while respecting the cultural diversity and uniqueness of the users. Such a process is needed if the population is different from the population in which the tool is originally developed (15, 18, 19). A previous study, as part of STrengthening Responses to Dementia In Developing countries (STRiDE), showed the importance of cross-cultural adaptation for maximizing cultural appropriateness and minimizing bias assessment instruments (20, 21).

The original BCSB was developed in Brazil and has been widely used and shown to be suitable in populations with various educational backgrounds (13, 14), as well as in homogenous population of sociocultural background and low educational levels (22). It has also been used in various clinical settings and showed a good ability to discriminate normal from cognitively impaired patients (23). Such a brief cognitive screening tool is urgently needed in a very diverse population such as Indonesia to improve the diagnostic and referral rates from primary healthcare facilities to memory clinics which are only available in certain urban areas in Indonesia. The BCSB was translated and adapted to Bahasa Indonesia, with the addition of phonemic fluency, and called BCSB-INA or *Instrumen Skrining Kognitif Ringkas Indonesia (INA-SKRin)* in Bahasa Indonesia to make it easier when administered in the population. Translation was applied to instruction and interpretation/scoring only, since it is a visual-based test. Using a visual-based cognitive assessment is a way to overcome the translational bias that usually comes from translating a verbal-based tool. The pre-testing result showed that it was generally accepted and understandable. The BCSB consists of the FMT and verbal fluency and CDT as the interference. The original pictures for the FMT were kept because they were considered to be universal.

### 4.2 Normative data and effect of sociodemographic factors

This study presents normative data from the feasibility study of 211 community-dwelling older adults in several urban and rural areas in North Sumatra, Indonesia. The interview was conducted by a number of trained interviewers at the primary healthcare facilities. The normative data presented here are similar to those in a previous study in Brazil, the country from which the BCSB was originally developed. We found the same median score for the FMT as the original, which was 8 for participants with education of more than 5 years and for those aged 65–74 years (24). This study found a lower score of the delayed recall subtest for people aged more than 75 years, which was 7. We also found that the FMT of the BCSB was not influenced by education. This finding indicates that the FMT can be used in older adult population with heterogenous educational backgrounds in Indonesia.

The FMT, particularly the delayed recall subtest of the BCSB (DR-FMT), has been widely used in epidemiological and clinical research and has shown high accuracy in the diagnosis of dementia across different levels of education (25). The influence of education on the FMT has been extensively studied in various subjects in population as well as clinical settings with a consistent finding of non-significant impact of education on the performance of the FMT (24, 26–28). This subtest of delayed recall has also been previously shown to have better performance than the Consortium to Establish a Registry for Alzheimer's Disease (CERAD) memory test in illiterate individuals (25, 29). The delayed recall subtest of BCSB has also been used in subjects with medium and high levels of education for dementia diagnosis with cut-off ≤ 5 and AUC 0.931 (30). Evaluation of cognitive abilities among older adults with limited education or those who are illiterate poses significant difficulties due to the fact that many cognitive assessment tasks within the battery presuppose a specific level of educational attainment (11). Our study shows that the younger age group (60–74 years) had better performance in several domains compared to those aged more than 75 years.

The fact that the BCSB is more influenced by age than education has also been shown by previous studies that found a correlation between age and DR-FMT (23, 24, 31).

Within the context of the BCSB, the CDT and verbal fluency serve as both a means of interference testing and a valuable tool for identifying cognitive impairment in individuals with moderate to high levels of education. However, its application in individuals with limited education is characterized by longer completion times and less information, likely due to the challenges these individuals face in completing paper-and-pencil visuospatial constructive tasks. Our study found that CDT and verbal fluency were influenced by education. This is in line with previous studies that showed older adults with low education had poorer performance on verbal fluency and CDT and significant influence of schooling for verbal fluency and CDT (23, 24, 26, 28, 32). Both semantic and fluency tests were influenced by age, education, and area of living with the scores being higher in younger age groups, higher education, and those living in urban areas. The results also showed that subjects living in urban areas showed better performance on learning, semantic and phonemic fluency tests, and CDT. Previous studies looking at the differences in cognitive function between people living in urban and rural areas showed that it can be attributable not only to differences in education but also to other factors. Some potential differences in cognition between older adults living in urban and rural areas include cognitive and social engagement, access to healthcare, environmental factors, and social factors (33, 34), although this needs to be further confirmed in future studies.

### 4.3 Implication for dementia care and practice in Indonesia

Dementia is characterized by a decrease in at least two domains of cognitive function or behavior that interferes with functional activities, which is not explained by a major psychiatric disorder or delirium, obtained from the history of the patient or from knowledgeable informants along with an objective cognitive assessment (35). The prevalence of dementia in Indonesia is projected to rise as the aging population increases. Therefore, the health system must be prepared to carry out an early and accurate diagnosis process in order to provide efficient dementia care. Early diagnosis is important for identifying potentially reversible causes, improving symptom management, and improving quality of life. In 2016, Indonesia launched a National Strategy on Management of Alzheimer's and Other Dementia Diseases: Toward Healthy and Productive Older Persons, which broadly covers the seven action areas agreed upon in the WHO's Global Action Plan on the public health response to dementia 2017–2025. It is meant to serve a purpose as a policy for collaborative actions to overcome the impacts of dementia. This comprehensive strategy includes the implementation of early detection, diagnosis, and holistic management of cognitive disorders and dementia (36). Key and major barriers to establishing timely diagnosis include the availability of cognitive tools suitable for the very diverse Indonesian population and the limited time of primary care physicians in conducting such assessments. Early symptoms of dementia also may not be apparent during an initial visit, unless they are assessed. Delayed and missed diagnosis of dementia leads to delayed and often missed opportunities for treatment that substantially increase the burden of care. The diagnosis of dementia mainly comes from clinical suspicion in patients presenting with cognitive complaints and/or changes in behavior (reported by the patients or their family), followed by cognitive and other examinations, such as laboratory or imaging, as indicated (35). The initial stage of dementia diagnosis mainly occurs in primary healthcare facilities. Therefore, improving the diagnostic algorithm in primary healthcare facilities is crucial not only for improved diagnostic accuracy but also for timely and higher referral rates to the appropriate healthcare facilities. Currently, in Indonesia, there are at least three different guidelines for screening tools for cognitive impairment in primary healthcare facilities, which have different clinical cut-off points. These include but are not limited to the Abbreviated Mental Test (AMT), the Minicog, CDT, and AD-8 (37–39). All these tools are available in Bahasa Indonesia, but there remain several implementation challenges, including the effect of education level, the limited cultural appropriateness, different conditions between urban and rural areas, the linguistic variability across different regions, and varying skills and time of the primary care physicians (PCPs) for carrying out these assessments. Our study found that using a visual-based test, such as the BCSB-INA, provides a way to perform cognitive screening that is free from bias of translation if the interviewer and the participants speak the same language. The short time to complete also makes this tool feasible to be administered in primary healthcare settings. However, it must be considered that the limited representation of adults over 75, coupled with a lower proportion of males in comparison to females, introduces a potential limitation to the robustness of the results in these demographic aspects. The smaller sample size of elderly individuals and the gender imbalance may impact the generalizability of findings, warranting caution in drawing broad conclusions from the study. Consideration of these demographic factors is essential for a comprehensive understanding of the results and their applicability across diverse age and gender groups. Further studies are needed to compare it with other cognitive and neuropsychology assessments and clarify its diagnostic accuracy in dementia.

## 5 Conclusion

In conclusion, the BCSB-INA, a cognitive screening battery adapted for use in Indonesia, demonstrates feasibility and efficacy in assessing cognitive function in older adults. Its adaptability to diverse education levels extends its utility, making it suitable for application in both urban and rural settings. The BCSB-INA's potential significance is underscored by its ability to provide culturally sensitive assessments, particularly crucial for the aging population in Indonesia's rural areas. Moreover, it is imperative to recognize that the interpretation of results should consider the norms established by this study. Results from the BCSB-INA may offer valuable insights into cognitive function among older individuals, but it is crucial to contextualize findings within the framework of the established norms. This consideration is especially pertinent when assessing individuals over 75 years of age. As we navigate the challenges associated with aging, the BCSB-INA emerges as a practical and adaptable tool for cognitive screening. Continued research further validates its role in providing nuanced assessments for diverse demographics, ultimately contributing to effective interventions and care strategies in these populations, particularly in the context of cognitive screening.

## Data availability statement

The raw data supporting the conclusions of this article will be made available by the authors, without undue reservation.

## Ethics statement

The studies involving humans were approved by Universitas Sumatera Utara Ethical Committee (approval number 498/KEP/FK USU/2022). The studies were conducted in accordance with the local legislation and institutional requirements. The participants provided their written informed consent to participate in this study.

## Author contributions

FF: Conceptualization, Data curation, Formal analysis, Funding acquisition, Investigation, Methodology, Project administration, Writing – original draft, Writing – review & editing. LN: Conceptualization, Methodology, Supervision, Writing – review & editing. YT: Writing – review & editing. DN: Writing – review & editing. AR: Writing – review & editing. RN: Writing – review & editing. PC: Writing – review & editing.
